# Solitary fibrous tumor of the prostate: Case report and literature review

**DOI:** 10.1016/j.eucr.2025.103207

**Published:** 2025-09-18

**Authors:** Alessandro Vengjer, Matheus Miranda Paiva, Eloi Guilherme Provinciali Moccellin, André Segura da Motta, Raphael Oliveira Emerick Constantino, Ana Luiza Tunes Ortiz, Katia Ramos Moreira Leite

**Affiliations:** a University Metropolitan of Santos (UNIMES), Brazil; b Santa Casa da Misericórdia of Santos, Brazil; c Faculty of Medicine of the University of Ribeirão Preto (UNAERP), Brazil; d Faculty of Medicine of ABC (FMABC), Brazil; e Faculty of Medicine of the University of São Paulo (USP), Brazil

**Keywords:** Prostate, Tumor, Fibrous, Solitary

## Abstract

Solitary fibrous tumor (SFT) of the prostate is a rare mesenchymal neoplasm with only a few cases reported. Its clinical presentation often mimics benign prostatic hyperplasia, and imaging findings are nonspecific. Diagnosis relies on histopathology and immunohistochemistry, particularly STAT6 nuclear expression. Most cases follow a benign course, but some may exhibit aggressive behavior. Complete surgical resection with clear margins is the treatment of choice, with no established role for adjuvant therapy. This report discusses a case of prostatic SFT, emphasizing its diagnostic challenges, imaging findings, and surgical management, contributing to the limited literature on this rare entity.

## 1 Introduction

Solitary fibrous tumor (SFT) is a rare neoplasm with an incidence of 0.61 to 0.37 per million people. Its classic location is the visceral pleura (80 %), while extrapleural tumors are distributed among the meninges, orbit, pancreas, liver, and peritoneum. In the urinary tract, the most commonly affected sites are the kidney and bladder.^1^ The prostate is an exceptionally rare site, with only 41 cases described in the literature, with the first case and the most recent ones cited in this paper (Table 1)^2^, ^3^, ^4^, ^5^, ^6^, ^7^, ^8^, ^9^, ^10^, ^11^, ^12^, ^13^, ^14^, ^15^, ^16^, ^17^, ^18^ representing less than 1 % of prostatic tumors.^13^ The median age at diagnosis in the largest series is between 50 and 60 years, ranging from 21 to 87 years. In general, there is no elevation in PSA levels, and the main symptom is lower urinary tract obstruction (LUTS) with eventual acute urinary retention, mimicking benign prostatic hyperplasia (BPH).

**Table 1:** Solitary fibrous tumor of the prostate: Literature review.

Author	Age (years)	PSA (ng/mL)	Outcome	Tumor Size (Specimen and Radiological)	Immunohistochemistry
Stolzenbach et al. (2023)	60	3,92	6 months without recurrence.	MRI: 25 × 31 × 27 mm	Not reported
Peng et al. (2022)	50	0.64	No recurrence after 3 months	Not described	Positive: CD34, Bcl-2, STAT6 Negative: CK
Matos et al. (2020)	66	0,4	5 years without recurrence	Not reported	Positive: CD34, BCL2 Negative:DOG1, CD117, S100, and Actine
Takeshima et al. (1997)	42	Not described	10 months - no recurrence	prostatectomy specimen weighed 720 g, and measured 14X13 × 11 cm	Positive: vimentin, CD34, Ki67 (4,5 %) Negative:cytokeratin, neuron specific enolase, myoglobin, prostatic acid phosphatase, estrogen receptor, progesterone receptor, DO-7 and PSA
Ahnou, D et al. (2021)	77	6.3	6 months without recurrence	MRI: Solid-cystic mass, 108 × 82 × 59 mm	Positive: CD34, Bcl-2, CD99 Negatives: Progesterone, AML, Pan-cytokeratin, S100, CD117
Gilbert et al. (2020)	78	2.0	12 months without recurrence	CT scan: Left peri-prostatic mass measuring 5.3 × 3.5 cm	Positive: CD34, Bcl-2, STAT6, Vimentin Negative: AE1/AE3, CD117, Cytokeratin
Feliu et al. (2022)	85	Not described	18 months without recurrence	polyglobulated (17 × 12 × 6 cm) mass partially covered by a fibrous capsule mass	Positive: CD34, Bcl-2, CD99, Vimentin Negative: EMA, Desmin, CKPAN, C-KIT, DOG-1
Liao et al. (2023)	67	3.22	No recurrence during the observed period	MRI: 6.8 × 6.4 × 8.2 cm Spherical tumor measuring 11 × 7.5 × 6 cm, weighing 223g	Positive: CD34, CD99, Vimentin, STAT6, Bcl-2 Negative: Desmin, S100, CK, ALK, CD117, Dog-1, SOX10
Wang et al. (2023)	64	1.268	12 months without recurrence	Post-surgical histopathological evaluation: 7 × 5 × 4 cm	Positivos: CD34, STAT6, BCL-2 Negativos: CD117, DOG1, S-100, SMA, SOX-10, Desmina Ki-67: 15 %
Heger et al. (2024)	76	1.2	Not described	transrectal ultrasound showed a prostate volume of 57.3 cc (before HoLEP)	Positive: Vimentin, STAT6, and Ki-67 (10–15 %) Negative: CD34, Cytokeratin, PSA, NKX3.1, CD10, PR, CD45, p63, Mart-1, SOX10, WT-1, Synaptophysin, S100, Smooth Muscle Heavy Chain
Yılmaz et al. (2023)	44	“Normal”	Approximately 1 cm additional growth was observed in the tumor (77 × 82 mm) in the 36th month CT images	MRI: 48 × 66-mm well-circumscribed mass	Positive: CD34, STAT-6, and vimentin Negative:CD99, bcl-2, and progesterone (PR), CD56, SMA, desmin, pancytokeratin, synaptophysin, CD31, S100, CD117, or DOG1
Takeuchi et al. (2021)	43	0.675	2 years without recurrence	3 × 3.4 cm (Tumor specimen)	Positive: STAT6 and CD34 Negative: Not described
Ronchi et al. (2017)	62	5.8	8 years without recurrence	Huge lobulated mass of about 20 × 10 cm involving the prostate gland (CT)	Positive: CD34, bcl2, CD99, STAT6 and partially for PgR Negative: CK), PSA, smooth muscle actin (SMA), calponin and CD117
Nishith et al. (2020)	54	Not described	Not described	(Ultrasound) 195.6g Pathology: Three nodular masses (5 × 4 × 3 cm, 4 × 4 × 3 cm, 4 × 3 × 2 cm)	Positive: vimentin, CD34, CD99 and BCL-2 Negative: pan-cytokeratin, epithelial membrane antigen (EMA), actin, desmin, myogenin, progesterone receptor (PR) and CD117
Yang et al. (2023)	57	Not reported	Not reported	Not reported	Positive: CD34, STAT6 and Ki67 (1 %)
Liu et al. (2019)	46	Not described	Pelvic recurrence in the 5th year after RP	MRI: 6.6 cm × 6.0 cm × 6.3 cm	Positive: CD34, CD99, B cell lymphoma-2, and Ki-67 Negative: progesterone receptor (PR), smooth muscle actin (SMA), tyrosine-protein kinase kit (CD117), cytokeratin-8/18 (CK8/18), and discovered on gist 1(DOG-1)
Eich et al. (2024)	51	1.13	12 months without recurrence	MRI: 2 cm T2 hypointense mass	Positive: CD34, BCL2, STAT6 Negative: Desmin, Smooth Muscle Actin (SMA)

The differential diagnoses include other mesenchymal tumors such as prostatic stromal tumor of uncertain malignant potential (STUMP), prostatic stromal sarcoma, rhabdomyosarcoma, schwannoma, gastrointestinal stromal tumor (GIST), inflammatory myofibroblastic tumor, leiomyoma, and leiomyosarcoma.^14^ Sarcomatoid carcinoma must also be ruled out. Immunohistochemical studies allow for diagnostic confirmation, as SFT typically expresses STAT6, CD-99, Bcl-2, and CD-34 while being negative for cytokeratins, actin, desmin, myogenin, CD-117, progesterone receptor, PSA, and S-100.^15^

Its etiology remains unknown; however, NAB2-STAT6 gene fusion is observed in most cases, leading to nuclear expression of the C-terminal portion of the signal transducer and activator of transcription 6 (STAT6), which is the most sensitive and specific marker. In general, SFT tends to have a benign course with slow growth, but recurrence and metastasis rates can reach up to 75 % in malignant tumors. The potential for malignancy is associated with tumor size (>10 cm), location, cellularity, nuclear atypia, mitotic activity, presence of atypical mitoses, and necrosis. Complete surgical resection with clear margins is crucial for increasing cure rates.^14^

The aim of this article is to report a case of solitary fibrous tumor of the prostate and review the literature on the subject.

## 2 Case report

**Fig. 1:**
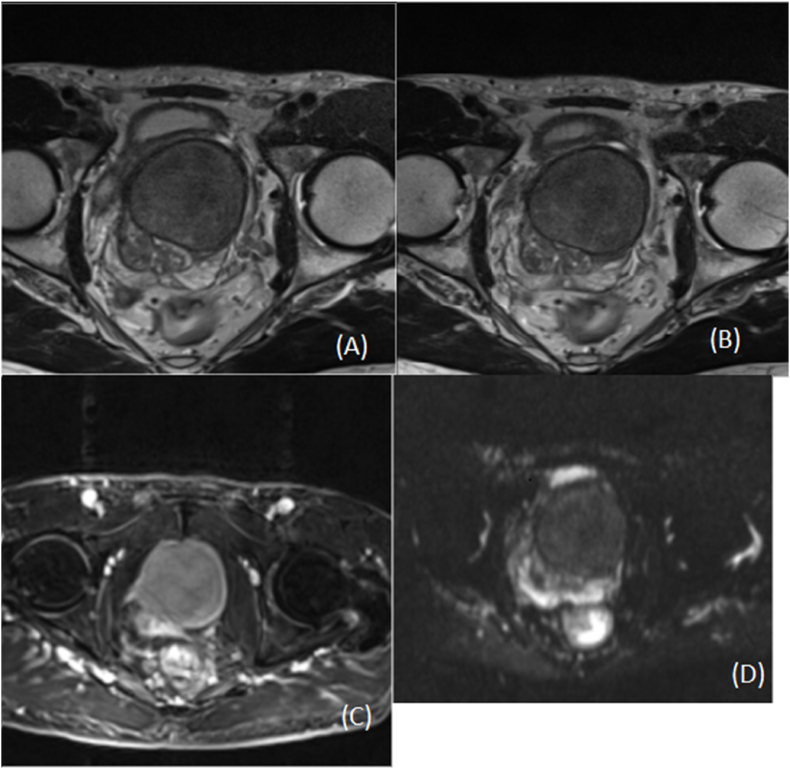
Magnetic resonance imaging showing an enlarged prostate (154.3 cc; 162 g) with superior bulging over the bladder floor. The peripheral zone presents normal diffusion and ADC values, with mildly heterogeneous T2 signal. The transition zone shows encapsulated nodules, with the largest located on the left (6.1 × 5.4 × 5.9 cm). Panel (A): anatomical image (T2-weighted sequence); panels (B) and (C): diffusion sequences; panel (D): perfusion.

A 70-year-old hypertensive, non-smoking patient sought medical attention due to LUTS complaints. He reported a sensation of incomplete emptying, nocturia three times per night, and a weak urinary stream. PSA levels were 4.22 ng/dL, with a free-to-total ratio of 24 %. Transrectal ultrasound (TRUS) showed a significantly enlarged prostate (222 g). Magnetic resonance imaging (MRI) (Fig. 1) revealed a 3.7 × 2.2 × 3.5 cm nodular lesion in the prostatic apex, classified as PI-RADS 2. Due to the clinical presentation, the patient underwent transvesical prostatectomy via robotic surgery.

**Fig. 2:**
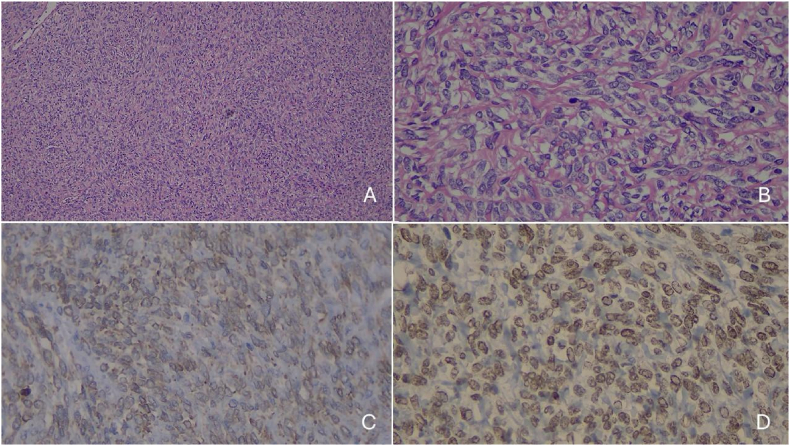
Histology showing a moderately cellular spindle cell neoplasm with mild atypia and low mitotic activity (Hematoxylin & Eosin - A: 40X; B: 400X). Immunohistochemical analysis demonstrates cytoplasmic expression of BCL-2 (C) and nuclear expression of STAT6 (D), characteristic of a solitary fibrous tumor.

Histopathological examination revealed a moderately cellular spindle-cell neoplasm with mild nuclear atypia, no mitotic activity, and no necrosis. Immunohistochemical analysis showed expression of Bcl-2, CD-34, and STAT6 (Fig. 2A and B) and was negative for smooth muscle actin, caldesmon, C-KIT, progesterone receptor, and S-100 protein (Fig. 2C and D), leading to the diagnosis of a solitary fibrous tumor.

**Fig. 3:**
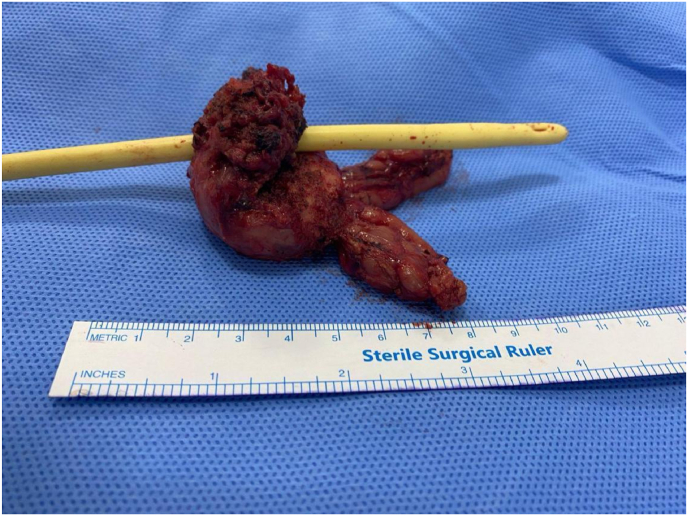
The radical robotic prostatovesiculectomy specimen revealed a residual tumor confined to an isolated nodule with an expansive growth pattern, displaying the same characteristics as the biopsy. The prostate weighs 38.5 g, measures 3.7 x 2.8 × 1.6 cm, and has a volume of 40 cc.

The patient had a good postoperative recovery, and due to the identification of a prostatic neoplasm, he underwent radical prostatovesiculectomy (with extended margins) via robotic surgery 30 days later for additional therapy (Fig. 3). The surgical specimen weighed 38.5 g (3.7 × 2.8 × 1.6 cm). A residual tumor measuring 5 mm was identified in the left anterior apex. The radial surgical margins were free of neoplasia, as were the bladder, urethral margins, and seminal vesicles. The patient had an uneventful postoperative course and remains asymptomatic, with preserved erectile function, continence, and undetectable PSA five months after surgery.

Informed consent was obtained from the patient for the publication of this case report and any accompanying images.

## 3 Discussion

Solitary fibrous tumor (SFT) of the prostate is a rare neoplasm, with 41 cases described in the literature (Table 1). The LUTS symptomatology and low PSA levels mimic BPH, and a definitive diagnosis is achieved exclusively through histopathological evaluation, complemented by immunohistochemical studies. MRI, which is currently used for the identification of clinically significant carcinomas, has low accuracy in detecting mesenchymal tumors, particularly rare neoplasms such as SFT. Reports of SFT in the literature have classified it as PI-RADS 3 to 5.^16^, ^17^, ^18^ In our case, MRI classified the lesion as PI-RADS 2, with a low probability of clinically significant carcinoma.

Histopathological diagnosis is challenging due to the need to differentiate SFT from other spindle-cell tumors that more commonly affect the prostate, such as STUMP, leiomyoma, leiomyosarcoma, rhabdomyosarcoma, malignant peripheral nerve sheath tumor, and monophasic synovial sarcoma. Sarcomatoid carcinoma must also be ruled out. The markers that allow differentiation among these tumors are listed in Table 2.^19^ The identification of NAB2-STAT6 fusion is specific to this neoplasm, and the detection of nuclear-localized protein by immunohistochemistry is specific to SFT.^14^

**Table 2:** Main differential diagnoses of prostatic solitary fibrous tumor and their immunohistochemical expression profiles.

Tumor	SMA	Caldesmon	CD34	STAT6	BCL2	Cytokeratins	S100	Myogenin	CD99	C-KIT	DOG1	PR	ALK1
**SFT**	_	_	+	+	+	_	_	_	+	_	_	_	_
**STUMP**	+/−	+/−	+	_	_	_	_	_	_	_	_	+	_
**PSS**	+/−	+/−	+	_	_	_	_	_	_	_	_	+	_
**SMT**	+	+	_	_	_	+/−	_	_	_	_	_	+/−	_
**GIST**	+/−	+/−	+	_	_	_	_	_	_	+	+	_	+/−
**RMS**	+	+	_	_	_	_	_	+	_	_	_	_	_
**SCH**	_	_	_	_	_	_	+	_	_	_	_	_	_
**IMT**	+	+	_	_	_	_	_	_	_	+/−	_	_	+/−
**CS**	_	_	_	_	_	+	_	_	_	_	_	_	_

CS – Sarcomatoid carcinoma.

GIST – Gastrointestinal stromal tumor.

RMS – Rhabdomyosarcoma.

SCH – Schwannoma.

PSS – Prostatic stromal sarcoma.

SMA – Smooth muscle actin.

STUMP – Stromal tumor of uncertain malignant potential.

SFT – Solitary fibrous tumor.

IMT – Inflammatory myofibroblastic tumor.

SMT – Smooth muscle tumors (leiomyoma/leiomyosarcoma).

SFT generally has a benign course, with malignancy potential indicated by lesion size, high cellularity, nuclear atypia, necrosis, mitotic activity >4/10 hpf, and the presence of atypical mitoses. A very low percentage of tumors exhibit aggressive behavior, usually with a tendency for local recurrence.^3^ One of the most important prognostic factors is complete surgical resection with clear margins. Since preoperative diagnosis is difficult to predict, surgical completion may occasionally be necessary, as in this case, where residual lesion was identified in the prostatic apex. There is no data supporting the indication of adjuvant therapy for this neoplasm.^17^

This study has certain limitations, including the rarity of SFTs, the retrospective nature of data, and the lack of long-term follow-up in many cases, which restricts broader conclusions about prognosis. However, it contributes to the growing body of literature by increasing the robustness of existing data and reinforcing key clinical patterns. Notably, this case highlights the diagnostic limitations of multiparametric MRI, where lesions may be assigned a high PI-RADS score—potentially suggesting malignancy—despite ultimately being benign. This underscores the importance of histopathological confirmation. For the patient, this has a direct impact by influencing surgical decision-making and avoiding overtreatment, while also raising awareness among surgeons for more accurate differential diagnoses.

## 4 Conclusion

SFT is a mesenchymal tumor with a challenging preoperative diagnosis, as it cannot be differentiated clinically, through laboratory tests, or by imaging methods such as multiparametric MRI, making it an unexpected pathological finding. It is still a rare condition with heterogeneous data in the literature, requiring more case reports for better understanding and diagnostic suspicion.

## CRediT authorship contribution statement

**Alessandro Vengjer:** Investigation, Writing – review & editing, Supervision, Formal analysis, Project administration, Conceptualization. **Matheus Miranda Paiva:** Funding acquisition, Resources. **Eloi Guilherme Provinciali Moccellin:** Resources. **André Segura da Motta:** Investigation, Writing – original draft, Project administration, Methodology, Supervision, Writing – review & editing, Conceptualization, Resources, Software, Validation, Formal analysis, Visualization. **Raphael Oliveira Emerick Constantino:** Visualization, Writing – original draft. **Ana Luiza Tunes Ortiz:** Writing – review & editing, Writing – original draft, Supervision, Project administration. **Katia Ramos Moreira Leite:** Visualization, Conceptualization, Project administration, Validation, Writing – review & editing, Formal analysis, Supervision, Data curation.
